# Microbial-Transferred Metabolites of Black Tea Theaflavins by Human Gut Microbiota and Their Impact on Antioxidant Capacity

**DOI:** 10.3390/molecules28155871

**Published:** 2023-08-04

**Authors:** Li Sun, You Su, Kaiyin Hu, Daxiang Li, Huimin Guo, Zhongwen Xie

**Affiliations:** 1 State Key Laboratory of Tea Plant Biology and Utilization, School of Tea and Food Sciences and Technology, Anhui Agricultural University, Hefei 230036, China; sljwc@ahtcm.edu.cn (L.S.); dxli@ahau.edu.cn (D.L.); huiminguo@ahau.edu.cn (H.G.); 2 The College of Pharmacy, Anhui University of Chinese Medicine, Hefei 230012, China; suyouahu@163.com (Y.S.); kaiyinhuaht@163.com (K.H.); 3 Center for Biotechnology, Anhui Agricultural University, Hefei 230036, China

**Keywords:** theaflavins, human gut microbiota, metabolites, UPLC-Q-Orbitrap-MS/MS, antioxidant capacity

## Abstract

Theaflavins (TFs), the primary bioactive components in black tea, are poorly absorbed in the small intestine. However, the biological activity of TFs does not match their low bioavailability, which suggests that the gut microbiota plays a crucial role in their biotransformation and activities. In this study, we aimed to investigate the biotransferred metabolites of TFs produced by the human gut microbiota and these metabolites’ function. We profiled the microbial metabolites of TFs by in vitro anaerobic human gut microbiota fermentation using liquid chromatography tandem mass spectrometry (LC-MS/MS) methods. A total of 17 microbial metabolites were identified, and their corresponding metabolic pathways were proposed. Moreover, full-length 16S rRNA gene sequence analysis revealed that the TFs altered the gut microbiota diversity and increased the relative abundance of specific members of the microbiota involved in the catabolism of the TFs, including *Flavonifractor_plautii*, *Bacteroides_uniformis*, *Eubacterium_ramulus*, etc. Notably, the antioxidant capacity of the TF sample increased after fermentation compared to the initial sample. In conclusion, the results contribute to a more comprehensive understanding of the microbial metabolites and antioxidant capacity of TFs.

## 1. Introduction

**Figure 1:**
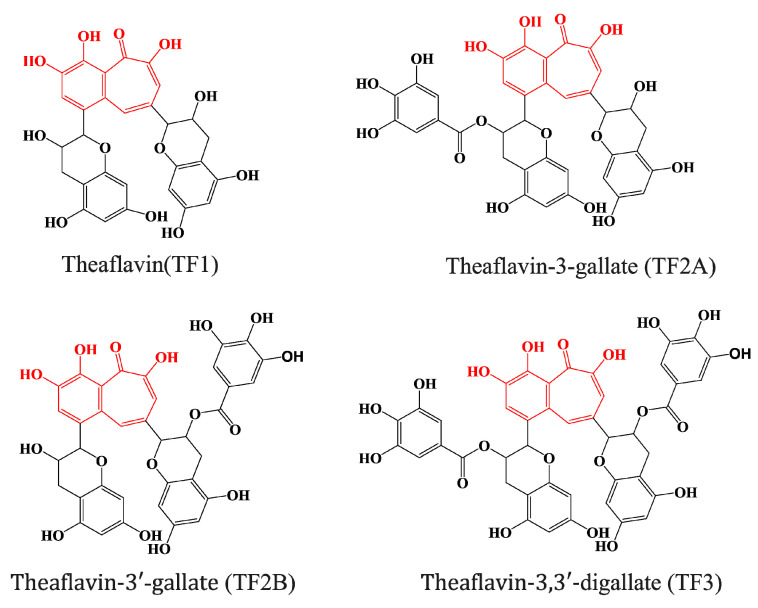
Chemical structures of major theaflavins in black tea. Red shading in the theaflavin structures highlights the same characteristic 1′,2′-dihydroxy-3,4-benzotropolone moiety.

Black tea, a type of oxidized tea, is the most consumed type of tea worldwide [1]. The consumption of black tea has been found to be associated with many health benefits, including the prevention of cancer and heart disease, which has been mainly attributed to the presence of polyphenolic compounds within the tea [2,3]. Theaflavins, including theaflavin (TF1), theaflavin-3-gallate (TF2A), theaflavin-3′-gallate (TF2B), and theaflavin-3,3′-digallate (TF3) [4], are the major bioactive polyphenols present in black tea. They possess a benzotropolone skeleton that is formed from the co-oxidation of selected pairs of catechins during oxidation (Figure 1) [5].

Recently, theaflavins have received extensive research attention due to their antioxidative [6], anti-inflammatory [7], anti-tumor [8], and antimicrobial properties.However, it has been reported that theaflavins have poor systemic bioavailability [9]. A major portion of unabsorbed TFs will reach the large intestine, where the gut microbiota will metabolize them to a wide range of lower-molecular-weight metabolites, which can then be absorbed in the colon [10]. In fact, the physiological effects of TFs cannot be fully achieved in the absence of microbial metabolites due to the extensive biotransformation of the parent compound. However, research on the biotransformed metabolites of TFs by gut microbiota is scarce. Therefore, it is crucial to profile the gut microbial metabolism of TFs.

The gut microbiota is believed to significantly contribute to the bioavailability of tea polyphenols [11], as well as their physiological functions, including their antioxidant [12], antiobesity [13], and anti-inflammatory activities [14]. In some cases, specific bacterial-transformation-produced metabolites have been found to increase their biological properties compared to the parent compounds. For example, it was reported that the catechin C-ring opening product, 1-(3′,4′-dihydroxyphenyl)-3-(2″,4″,6″-trihydroxyphenyl)propan-2-ol, exhibited a higher antioxidant activity than intact catechin [15]. Furthermore, recent studies have highlighted the critical role of these microbial metabolites in the overall bioactivity of polyphenols [1,16]. Therefore, investigating the biotransformation of TFs by human gut microbiota is essential for evaluating the actual health-promoting effects of TFs. 

In vitro anaerobic fermentation is a practical method for investigating the gut-microbiota-mediated metabolism of polyphenols [17,18,19]. To date, several studies have reported on the gut microbial metabolism of TFs [10,20,21]. However, information concerning the microbial metabolites of TFs in the human gut remains limited. A comprehensive characterization of the microbial metabolism of TFs is crucial for understanding their physiological effects and mechanisms in humans, owing to the significant contribution of microbial metabolites to overall bioactivity. In this study, a rapid and efficient method was established to identify and characterize TF metabolites. Then, the microbial metabolites of the TFs were investigated using in vitro anaerobic fermentation combined with LC-MS/MS methods, as well as the dynamics of the microbiota during fermentation using full-length 16S rRNA. The impact of the microbial metabolites on the TFs’ antioxidant capacity was also evaluated. The obtained results provide a better understanding of the microbial metabolites of TFs in the human gut and offer insight into the essential role microbial metabolites play in the overall bioactivity of TFs that are related to health benefits.

## 2. Results

### 2.1. Mass Fragmentation Behavior Analyses of TFs

**Figure 2:**
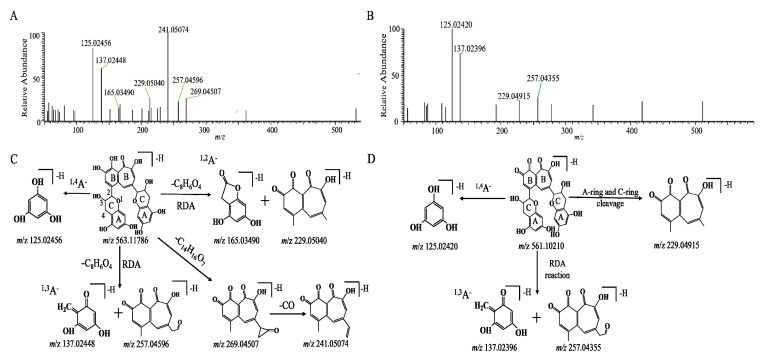
The MS/MS data and possible mass spectrometric cleavage pathway of TF1 (**A**,**C**) and the TF metabolite theaflavin quinone (**B**,**D**).

The chromatographic and mass spectrometric behaviors of the TFs were investigated by UPLC-Q-Orbitrap MS/MS in negative electrospray ionization (ESI) mode. Taking deprotonated TF1 as an example, the deprotonated molecular ion of TF1 at *m*/*z* 563.11816 with the chemical formula of C_29_H_24_O_12_ was observed at 11.28 min. The MS/MS spectrum of TF1 is shown in Figure 2. In the spectrum, two fragment ions at *m*/*z* 269.04272 and 241.05058 were obtained after the loss of C_14_H_16_O_7_ and CO, respectively. The fragment ion at *m*/*z* 241.05 may have been formed by the fragment ion of the benzotropolone moiety, which was observed in the MS/MS fragmentation of TF1, TF2A, and TF2B (Figure S3). Moreover, the product ions at *m*/*z* 137.02448, 257.04596, 165.03490, and 229.05040 were generated via retro-Diels–Alder (RDA) fragmentation of the flavan-3-ol heterocyclic A-ring. Meanwhile, the ion at *m*/*z* 125.02456 was formed after the 1,4 bonds of the C-ring (^1,4^A^−^) were broken. These ions may be considered diagnostic product ions for theaflavins (TFs) and their derivatives. The proposed mass fragmentation patterns of TF1 are illustrated in Figure 2C.

### 2.2. Metabolites of TFs by In Vitro Human Fecal Fermentation

The metabolic fate of the TFs during their fermentation for 48 h with the human gut microbiota was monitored by the established UPLC-Q-Orbitrap-MS/MS analysis method using the full mass/dd-MS2 scan mode, which belonged to the data-dependent acquisition (DDA). The potential metabolites were identified through a comparison with the two controls (see details in Section 4.4 and Supplementary Figure S1). In this study, we selected the potential metabolites of the TFs based on strict standards: either ions that appeared in the TF group but did not appear in the control groups, or ions whose response strength differed by more than five times. Herein, a total of 17 potential metabolites of the TFs were identified, 9 of which were confirmed with authentic standards. All of these metabolites, except for 3-hydroxyphenylacetic acid and 4-hydroxybenzoic acid, were absent from the blank samples. These two exceptions were also identified in the control samples, in which they may have been derived from other food phenolics but at a much lower relative abundance (less than 20%), compared to the TF-fermented samples. Their corresponding retention times, tentative identifications, molecular formulas, detected mass-to-charge ratios (*m*/*z*), and corresponding mass errors (Δ*m*/*z*), as well as the characteristic MS/MS fragment ions, are shown in Table 1 and Figure S3.

Based on their chemical structures, the metabolites can be classified into two categories, namely TFs and derivatives, and phenolic catabolites. Compounds in the class of “TFs and derivatives” contained their dimeric structure. A total of eight TFs and derivatives were characterized after the fecal fermentation. The peak areas of M1, M2, and M3 in the 2 h fermentation sample showed more than a five-fold increase compared to those in the 0 h unfermented sample, suggesting that they were formed from TF3 via degalloylation by the fecal microbiota. Based on the corresponding reference standards, M1, M2, and M3 were identified as TF2A, TF2B, and TF1, respectively. The cleavage of ester bonds was further confirmed by the identification of gallic acid (M9) and pyrogallol (M10), the decarboxylation product of gallic acid, through a comparison with authentic standards. For the other metabolites, due to the unavailability of corresponding commercial standards, they were identified through a comparison with the data obtained from the mentioned reference compounds and the published literature.

As shown in Figure 2D, the molecular formula of M4 was C_29_H_22_O_12_, and its molecular ion was [M − H]^−^ with an *m*/*z* of 561.10266 and a molecular weight that was 2 Da (H_2_) less than TF1. From the high-resolution mass spectra obtained by UPLC-HRMS, we speculated that the ion with an *m*/*z* of 229.04915 was produced from the loss of an A-ring and a C-ring. In addition, similar to TF1, both peaks had the typical fragment ions of an A-ring at *m*/*z* values of 125.02420 and 137.02396, respectively, from the fragmentation of the A-ring. We hypothesized that its structure was similar to theaflavin (563.11932, C_29_H_24_O_12_), potentially corresponding to a derivative in which H_2_ is lost via the oxidation of the six-membered ring of the benzotropolone moiety. Based on the molecular structure, molecular weight, and mass spectral cleavage pathway of the prototype TF1, we tentatively suspected that M4 might be the theaflavin quinone (TQ).

M5 presented [M − H]^−^ at an *m*/*z* of 533.10876, indicating a molecular formula of C_28_H_22_O_11_ (Δ*m*/*z* = −2.97 ppm). The ESI-MS^2^ spectrum of this compound presented six major fragment ions with *m*/*z* values of 349.07263, 125.02444, 137.02464, 165.01924, 377.06494, and 241.05017, respectively, which were in accordance with the spectral data previously reported for the anaphthoquinone (TNQ) by Liu et al. [22]. The absence of a deprotonated ion corresponding to gallic acid (*m*/*z* of 169) means that there are no galloyl moieties in the chemical structure of M5, which is proposed to be a metabolite of TF1. Therefore, M5 was tentatively identified as TNQ based on the mass spectral cleavage pathways (Figure S2) and the reported literature [23].

M6 and M7 exhibited the same molecular ion at an *m*/*z* of 565 with a molecular formula of C_29_H_26_O_12_, which is two Da (H_2_) greater than that of TF1. A previous study reported that the gut bacteria *Eggerthellalenta* (*Eggerth*) possess the ability to cleave the C-ring of TF1 [15]. Furthermore, analogous to TF, both peaks displayed the characteristic fragment ions of the A-ring at an *m*/*z* values of 125.02444 and 137.02446, respectively, resulting from the fragmentation of the A-ring, and the fragment ion of the benzotropolone moiety at an *m*/*z* of 241.05017. Therefore, based on the proposed molecular formulas and the MS/MS fragmentation data, M6 and M7 were tentatively identified as the C-ring cleavage metabolites of TF1 (DH-TF). Considering their Clog *p* values (−0.987216 and −0.987217, respectively), they were further tentatively identified as DH-TF1 and DH-TF2, respectively. Since there were two C-rings present in the TF1 structure, M8 was extracted in the extracted ion chromatogram (XIC) at 8.70 min and detected at an *m*/*z* of 567.14929, which is four Da(H4) less than that of TF1; it was proposed to be a double C-ring cleaved metabolite of TF (TH-TF). However, the content of M8 was too low to acquire the corresponding MS2 spectrum.

Phenolic catabolites are downstream metabolites that result from the ring fission of flavonoid skeletons. As illustrated in Table 1, a total of nine phenolic catabolites were detected in the TF fermentation samples. M11 and M12 shared the same molecular formula of C_11_H_12_O_4_ and yielded deprotonated ions with *m*/*z* values of 123.04528, 163.07660, and 122.03704, respectively. Considering their Clog *p* values (−0.0500000 and −0.019999, respectively), they were further tentatively identified as (3′,5′-dihydroxyphenyl)-γ-valerolactone and 5-(3′,4′-dihydroxyphenyl)-γ-valerolactone, respectively.

M13 (C_11_H_14_O_4_, error = −3.09 ppm) was eluted at 9.81 min and exhibited MS2 fragments at *m*/*z* values of 123.08167,147.08159, 165.09203, and 191.07146. Owing to the consistency of these data with those reported in the literature [15,22], M12 was tentatively identified as either 5-(3′,4′-dihydroxyphenyl) valeric acid or 5-(3′,5′-dihydroxyphenyl)valeric acid. Further methodologies are necessary to determine the exact binding sites for the hydroxyl moiety in these compounds. Compared with authentic standards, M14, M15, M16, and M17 were unequivocally identified as 3-(3′,4′-dihydroxyphenyl)propanoic acid, phenylacetic acid, 3-hydroxyphenylacetic acid, and 4-hydroxybenzoic acid, respectively. Due to the presence of the carboxyl group, these phenolic catabolites all generated characteristic product ions with a neutral loss of CO_2_ (44 Da) [24,25].

**Figure 3:**
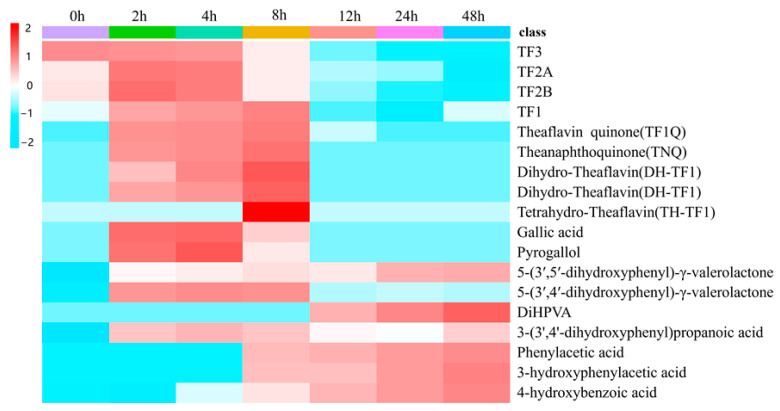
Heat map of dynamic changes in the peak areas of microbial TF metabolites at different fermentation times of 0, 2, 4, 8, 12, 24, and 48 h. Red and blue boxes represent values that are higher and lower than the mean value, respectively.

**Figure 4:**
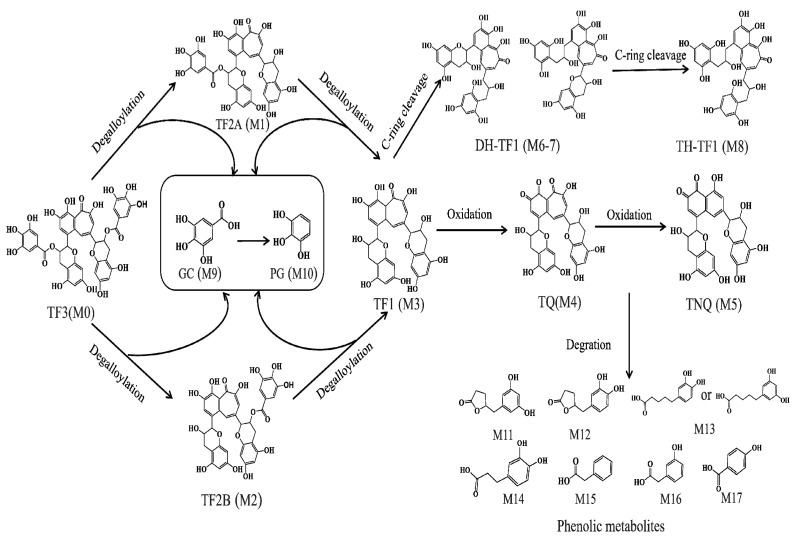
Potential pathways for the metabolism of TFs by human gut microbiota. The numbers of metabolites correspond to those in Table 1.

In addition, this experiment was designed to detect the metabolites of TFs by human fecal fermentation at different time points in order to describe the dynamic changes that occurred in the compounds. A heat map of the dynamic changes that took place in the peak areas of 17 TF metabolites is shown in Figure 3. As depicted in Figure 4, a rapid degradation was observed for TF3. After 12 h of fermentation, TF3 was almost entirely degraded. Conversely, the peak areas of TF1, TF2A, and TF2B showed a trend of rising first and then decreasing during the fermentation process, particularly that of TF1, which reached its maximum level at 8 h and subsequently declined significantly; this may suggest that TF3 may be metabolized by the gut microbiota into TF1, TF2A, and TF2B. The metabolites of the TF derivatives were detected first at 2 h, and they were found to increase from 2 h to 8 h (TQ, TNQ, etc.). In addition, some phenolic catabolites were initially detected at 12 h, implying that TFs may degrade via C-ring cleavage and oxidation by the gut microbiota, ultimately resulting in smaller phenolic metabolites. In addition, the peak areas of all identified metabolites detected fermentation broth at different fermentation times of 0, 2, 4, 8, 12, 24, and 48 h (Table S1).

### 2.3. Biotransformation Pathway of TFs by In Vitro Human Fecal Fermentation

In this study, the microbial-mediated degradation of TFs resulted in an array of metabolites. Based on the identified metabolites listed in Table 1, the TFs underwent a series of microbial-mediated biotransformations and the initial steps of metabolism, including sequential ester hydrolysis, C-ring cleavage, and oxidation. These reactions led to the formation of upstream metabolites, including DH-TF1, TH-TF2, TQ, and TNQ. Subsequent degradation reactions then occurred, including A-ring fission, dehydroxylation, and aliphatic chain shortening, which produced downstream metabolites comprising several phenolic metabolites. The proposed biotransformation pathways of the TFs during in vitro human fecal fermentation are illustrated in Figure 4.

### 2.4. Dynamics of Microbiota during Fermentation Process

**Figure 5:**
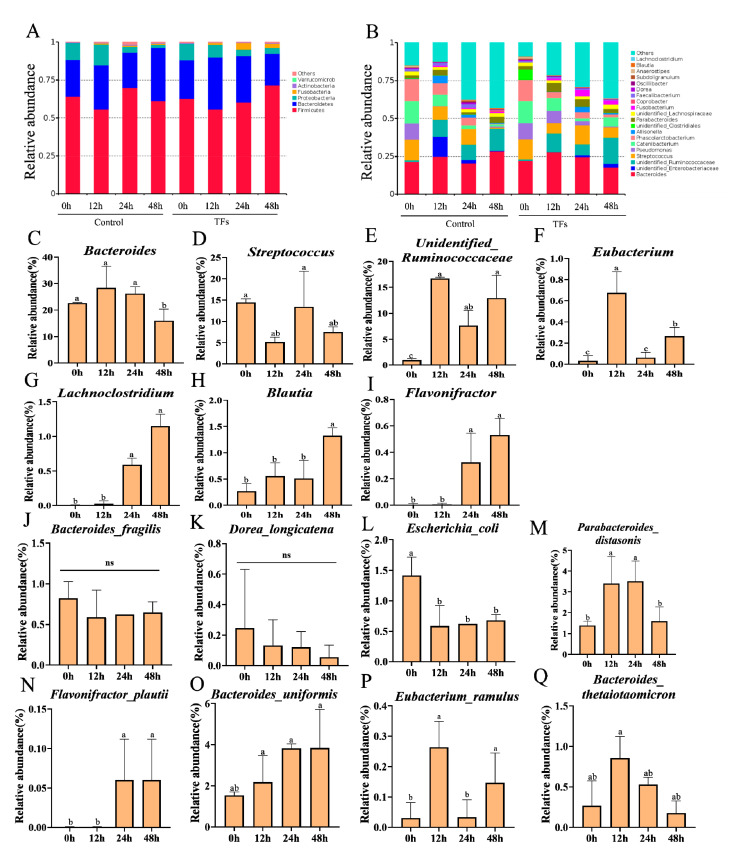
Dynamics of microbiota during fermentation process at different time points of 0, 12, 24, and 48 h. The relative abundances of gut microbiota at phylum level (**A**) and at genus level (**B**). The specific dominant members of the gut microbiota with the ability to catabolize flavan-3-ols and their relative abundances at the genus level (**C**–**I**). The dominant gut microbiota with ability to catabolize flavan-3-ols on the relative abundances at the species level (**J**–**Q**). Data are presented by the mean ± SD (n = 3). Different letters above group columns indicate a significant difference (*p* < 0.05).

The microbiota community compositions in the fermentation samples at 0, 12, 24, and 48 h were assessed using the Illumina high-throughput sequencing of bacterial 16S rRNA genes. After merging and filtration were carried out, a total of 183,645 reads were obtained for the microbiome analysis across all the samples. After taxonomic annotation was performed, the relative abundances of these operational taxonomic units (OTUs) in each sample were used for further analysis. In general, sequences with at least 97% similarity were clustered into OTUs, which can represent community richness. These OTUs were assigned to six phyla, namely *Bacteroidetes*, *Firmicutes*, *Proteobacteria*, *Fusobacteria*, *Actinobacteria*, and *Verrucomicrobia* (Figure 5A). At the genus level, a total of 70 bacterial genera were identified across all the samples. The changes in the average relative abundances of the 25 most abundant genera during the fermentation process are shown in Figure 5B. In the TF samples, as well as in the control samples, a decrease in the *Bacteroides* and an increase in the *unidentified_Ruminococcaceae* were observed. However, it was found that the TFs had significantly increased the relative abundances of the *unidentified_Ruminococcaceae*, *Lachnoclostridium,* and *Flavonifractor* compared to the control after 48 h of fermentation (*p* < 0.05). Thus, the supplementation of the TFs for 48 h significantly affected the microbiota composition. The comparative analysis revealed that the microbiota composition of the TF sample at 0 h was significantly different from that of the TF sample at 48 h. *Bacteroides* and *Streptococcus* are two of the major genera of bacteria that we measured at the different fermentation time points of 0, 12, 24, and 48 h (Figure 5C,D). Members of the microbiota with the ability to catabolize flavan-3-ols, including *Eubacterium*, *unidentified*_*Ruminococcaceae*, *Lachnoclostridium*, *Blautia*, and *Flavonifractor*, showed a dramatic increase with the fermentation of the theaflavins (TFs), particularly in the TF sample at 48 h compared to the TF sample at 0 h (*p* < 0.05) (Figure 5E–I) (*p* < 0.05). Most notably, we found that the relative abundance of the *Eubacterium* reached its maximum in the TF sample at 12 h. At the species level of microbiota (Figure 5J–Q), the fermentation of the TFs significantly increased the relative abundances of the *Parabacteroides_distasonis*, *Flavonifractor_plautii*, *Bacteroides_uniformis*, *Eubacterium_ramulus*, and *Bacteroides_thetaiotaomicron*, indicating that TFs exert a selective proliferative effect on gut microbiota and that they can be simultaneously catabolized.

### 2.5. Antioxidant Activity of TF Fermentation

**Figure 6:**
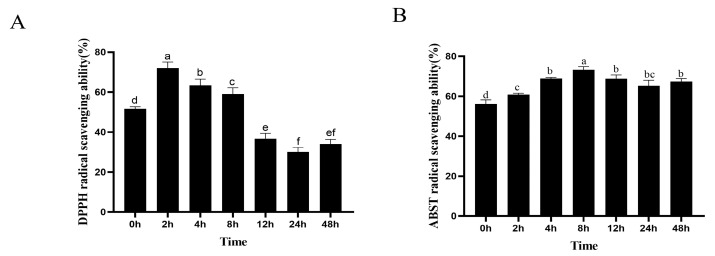
The dynamic changes in DPPH radical scavenging abilities (**A**) and ABTS radical scavenging abilities (**B**) of TFs after fermentation with human intestinal microbiota. Data are expressed as mean values, with their SDs (n = 3) depicted by vertical bars. Different letters above group columns indicate a significant difference (*p* < 0.05). The “ef” above 48 h column denotes a lack of significant difference when compared to the “e” group of the 12 h column, and “f” group of 24 h column (**A**). The “bc” above 24 h column indicates a lack of significant difference when compared to the “b” group of the 4 h, 12 h, and 48 h columns, and “c” group of the 2 h column, respectively (**B**).

To reveal the function of antioxidant activity during the biotransformation process of TFs by human fecal microflora, the TFs’ DPPH and ABTS radical scavenging capabilities were examined at seven time points. The results are depicted in Figure 6. The fermented TF samples at 2, 4, and 8 h exhibited a significant increase in DPPH radical scavenging activities (*p* < 0.05), especially at the 2 h time point. In other words, the fermentation-associated microbial metabolites exhibited a higher antioxidant activity than the original unfermented TFs. Most notably, the TF samples after fermentation also showed the highest ABTS radical scavenging activities (*p* < 0.05). However, due to the different mechanisms and reaction times of the assay methods, the measured DPPH and ABST radical scavenging capability values exhibited slight differences.

## 3. Discussion

Theaflavins (TFs) are one of the primary functional phytochemicals found in black tea, contributing to the unique flavor of specific teas and providing health benefits [7,26]. According to recent research [12,27,28], TFs demonstrate antimicrobial, hypolipidemic, anti-inflammatory, anti-mutagenicity, and anti-cancer activities. As orally administered natural products, TFs have low bioavailability. However, information on their metabolic fate in the human gut by microbiota is very limited, and the effective components of TFs may not be limited to the prototype substance itself.

An increasing body of research has indicated that the metabolites produced through the biotransformation of tea polyphenols by the gut microbiota contribute significant health benefits. Therefore, investigating the metabolites of TFs produced by the human gut microbiota is of paramount importance. To date, the gut-microbiota-mediated metabolism of TFs has been investigated in several studies. Using the HPLC method, Chen et al. analyzed the degradation profile of TFs co-incubated with the feces of healthy individuals, revealing that TF3 was extensively metabolized into TF2A, TF2B, TF, GA, and PG, which corroborates our findings [21]. Further, over time, they found that the concentration of TF1 in the incubation system progressively diminished, indicating that the potential further degradation of TF1 occurred. However, the respective downstream metabolites remained unidentified [21]. Pereira-Caro et al. explored the gut microbial metabolism of TFs by utilizing liquid chromatography tandem mass spectrometry (LC-MS/MS) technology, and they identified the further degradation of TFs into 3-(4′-hydroxyphenyl) propionic acid [10]. In a separate study, Liu et al. investigated the metabolic profile of TF3 using LC-MS/MS, identifying nine microbial metabolites. Further, they found that TF1, formed by the degalloylation of TF3, can be oxidized to TNQ by human gut microbes, a finding which supports our results [22]. However, information concerning the comprehensive microbial metabolism of TFs in the human gut microbiota remains very limited.

In this study, we investigated the gut microbiota-mediated metabolic characteristics of TFs. Thanks to our use of the growing number of available reference standards and the established UPLC-Q-Orbitrap-MS/MS analysis method, we identified a total of 17 potential metabolites in the TF fermentation samples, a considerably higher number than in previous research, with this result highlighting the pivotal role which the human gut microbiota plays in the biotransformation of TFs. The structures and mass spectral fragmentation patterns of these potential metabolites were analyzed using an UPLC-Q-Orbitrap-MS/MS system. Four metabolites (M4, M6, M7, M8) were confirmed in the human gut microbiota. It can be postulated that certain gut microbes may possess the enzymatic capability to oxidize TF1 into its quinone form (M4), which may subsequently undergo further oxidation to generate TNQ (M6). However, the specific biotransformation pathways involved in this process warrant further investigation. Based on these metabolites, we proposed more comprehensive microbial-mediated TFs metabolic pathways, as illustrated in Figure 4. Concurrently, we delineated the temporal relationships among all the metabolites, contributing to our understanding of TFs metabolism mediated by intestinal bacteria.

Growing evidence indicates that the metabolic pathways of dietary polyphenols are closely associated with the gut microbiota. In this study, we observed that TF supplementation considerably increased the diversity of the modulatory gut microbiota. Previous research has reported that specific *Bacteroides* species are implicated in the biotransformation of flavonoids [27]. For instance, rutin can be hydrolyzed by the α-L-rhamnosidase enzyme produced from *Bacteroides* sp. 54 into quercetin 3-*O*-glucoside, quercetin, and leucocyanidin. Furthermore, Quercitrin is also degraded to quercetin by *Bacteroides* sp. 45 and subsequently undergoes ring cleavage to yield 3,4-dihydroxybenzoic acid [28]. Additionally, in vitro co-culture studies have demonstrated that tannic acid is degraded by tannase from *Streptococcus gallolyticus* subsp. *Gallolyticu*s (S.G.G.) to produce pyrogallol [29]. In our investigation, *Bacteroides* and *Streptococcus* constituted two of the predominant bacterial genera measured. *Ruminococcus*, isolated from human feces, can cleave the C-ring of quercetin to generate a series of small phenolic acid compounds [30]. In our study, the abundance of *unidentified_Ruminococcus* was found to have significantly increased after the fermentation process was carried out. Moreover, *Eubacterium*, one of the most extensively researched flavonoid-degrading gut bacteria, is a core genus of the human gut microbiota. It has been reported that flavanone-/flavanonol-cleaving reductases from *E. ramulus* degrade specific flavonoids, yielding chalcone and dihydrochalcone [31]. Our study found that the TFs substantially elevated the relative abundance of *Eubacterium*, particularly at the transformation time of 12 h. Similarly, research has demonstrated that *Blautia*, *Enterococcus*, *Lachnoclostridium*, and *Flavonifractor* possess the capability to participate in the metabolism of flavonoids, subsequently generating small phenolic acids through the cleavage of the C-ring of TF derivatives [26,30]. Thus, these specific intestinal bacteria may promote the metabolism of compounds in TFs. At the species level of the microbiota, our study found that the supplementation of TFs significantly increased the relative abundances of *Parabacteroides_distasonis*, *Flavonifractor_plautii*, *Bacteroides_uniformis*, *Eubacterium_ramulus*, and *Bacteroides_thetaiotaomicron*. These members of the microbiota have been reported to catabolize flavan-3-ols compounds, suggesting that they may be involved in the biotransformation of TFs [31]. However, the distinct microbial species that contribute to specific phenolic metabolism processes warrant further investigation.

To assess the impact of human gut microbiota fermentation on the functional activity of TFs, we conducted an antioxidant activity evaluation. The study found that, compared to the pre-fermentation TF samples at 0 h, the post-fermentation TF samples exhibited an enhanced antioxidant activity, indicating that TF metabolites possess significant antioxidant properties. Recent studies have shown that the catechin C-ring opening product 1-(3′,4′-dihydroxyphenyl)-3-(2″,4″,6″-trihydroxyphenyl)propan-2-ol exhibits higher antioxidant activity than intact catechin [15]. Thus, we speculated that the TF1 C-ring opening product (M6–M8) had a higher antioxidant activity than intact TF1. Nonetheless, due to this study’s limitations, further investigation needs to be conducted. In addition, the microbial metabolites of the TFs (M4, M5, M9, and M10) exhibited substantial antioxidant properties, showing larger peak areas between 2 and 8 h of fermentation. Here, we speculated that these metabolites may have contributed to the increased antioxidant activity of the TFs observed during fermentation from 2 to 8 h. However, the individual metabolites of TFs need to be further explored in the near future.

## 4. Materials and Methods

### 4.1. Chemicals

Theaflavins (TF), theaflavin-3-gallate (TF2A), theaflavin-3′-gallate (TF2B), and theaflavin-3,3′-digitate (TF3), all with purities of 98%, were purchased from Chengdu Munster Biotechnology Co., Ltd. (Chengdu, China). Gallic acid, pyrogallol, 4-hydroxybenzoic acid, 3-hydroxyphenylacetic acid, phenylacetic acid, and 3-(3′,4′-dihydroxyphenyl) propanoic acid, all with purities of at least 98%, were purchased from Shanghai Yuanye Biotechnology Co., Ltd. (Shanghai, China). 

2,2′-azino-bis-(3-ethylbenzothiazoline-6-sulfonic acid) (ABTS), 2,2-diphenyl-1-picrylhydrazyl (DPPH), and 2,4,6-Tris(2-pyridyl)-s-triazine (TPTZ) were purchased from Sinopharm Chemical (Shanghai, China). Acetonitrile (LC/MS grade) was obtained from Merck (Darmstadt, Germany). Formic acid (LC/MS grade) was purchased from Fisher Scientific (Loughborough, UK). Deionized water (18.2 MΩ/cm) was prepared by distilling water through a Milli-Q water purification system (Millipore, Billerica, MA, USA). Other reagents were all of analytical grade.

### 4.2. Sample Preparation

In this study, theaflavins (TFs) consisting of four theaflavin monomers (TF1, TF2A, TF2B, and TF3) whose content percentages were 15.23%, 35.42%, 33.24%, and 16.11%, respectively, were used to reproduce those found in Keemun black tea, based on the data obtained for the primary theaflavins in Keemun black tea from prior experiments [32]. The TFs sample was diluted in sterile ultrapure water before being employed for in vitro fermentation, resulting in a concentration of 0.1 mg/mL for later use.

### 4.3. Preparation of the General Anaerobic Medium Broth

General anaerobic medium (GAM) broth was prepared according to previous publications [33]. Briefly, 30 g of GAM was dissolved in 1000 mL of distilled water, and the pH value was adjusted to 7.0. After being treated with an anti-bacterial process with high pressure (0.15 MPa) and high temperature (121 °C) for 20 min, the cooled GAM broth was then transferred to an anaerobic chamber (37 °C, anaerobic condition), in which 1 mg of vitamin K1 and 6 mg of hematin chloride were added to it.

### 4.4. Fecal Sample Collection and In Vitro Fermentation of TFs with Human Gut Microbiota

The in vitro fecal fermentation of TFs was performed following the methodology described by Liu et al. [22] with some modifications. Fresh human feces were collected from 8 healthy Chinese volunteers (18–24 years; 4 males and 4 females). All volunteers reported no consumption of tea in the week prior to the donation, were in good health, and had not been given antibiotics for at least 3 months before the collection. All the fecal materials were stored at −80 °C immediately.Equal amounts of fecal materials (1.0 g) from the eight volunteers were transferred to an anaerobic chamber (5%H_2_, 10% CO_2_, and 85% N_2_) to thaw for 3 h at 37 °C. After they thawed, fecal slurries were prepared by mixing fresh feces samples with autoclaved DPBS (0.1 M, pH 7.2) to yield a 10% (*w*/*v*) suspension, and this was further strained through four layers of cheese cloth to obtain a homogeneous human fecal suspension (HFS). Then, the filtered HFS (2.7 mL) was added to GAM broth (24.3 mL) and was incubated at 37 °C in the anaerobic chamber for 12 h in order to activate the bacteria. Subsequently, 3 mL of sterile TFs was added to the suspension (27 mL), with the final concentration of TFs being 0.1 mg/mL. As a control, 3 mL of water was added to 27 mL of HFS. The mixtures were then incubated at 37 °C in the anaerobic chamber. The TF samples were collected at seven time points (0, 2, 4, 8, 12, 24, and 48 h) of fermentation and immediately diluted in 3 mL of pre-cooled ACN in order to stop the fermentation. After centrifugation was carried out (30 min, 12,000 rpm, 4 °C), the supernatants were collected for the analysis of TFs and their metabolites by LC-MS/MS and measurement of antioxidant activity. For gut microbiota analysis, 3 mL of the samples at fermentation times of 0, 12, 24, and 48 h were collected and immediately frozen at −80 °C until bacterial DNA extraction. All experiments were repeated in triplicate. 

In this experiment, we used the mixed TF samples plus human fecal suspension (HFS) with GAM medium fermented at 0, 2, 4, 8, 12, 24, 48 h, respectively, as the experimental group. Time point at 0 sample as control was set to exclude TFs interacted with HFS in both the transferred metabolites and microbiota analysis. At the same time, water (TF replacement) and HFS with GAM medium fermented at 2, 4, 8, 12, 24, 48 h, respectively, were also used as control to exclude the interference from fecal suspension and culture medium components.

### 4.5. Identification of the Microbial Metabolites of TFs by UPLC-Q-Orbitrap-MS/MS

To identify the microbial metabolites of TFs, the obtained supernatants were analyzed using a UPLC system (Thermo Fisher Scientific, Waltham, MA, USA) equipped with a binary pump solvent management system with an online degasser, autosampler, and column oven. The samples were separated on an Acquity UPLC BEH C18 column (100 mm × 2.1 mm, 1.8 μm; Waters, Milford, MA, USA), and the column temperature was maintained at 40 °C. The injection volume was 2.0 μL. The mobile phases, consisting of A (0.75% formic acid in water) and B (0.75% formic acid in CAN), were used at a flow rate of 0.3 mL/min. The elution program was set as follows: isocratic at 1% (*v*/*v*) B for 2 min; 2–22 min linear gradient to 99% (*v*/*v*) B; 22–25 min isocratic at 99% (*v*/*v*) B. The mobile phase was adjusted to starting conditions for 1 min, followed by equilibration for 4 min.

For mass spectrometric analysis, a Thermo Q Exactive Focus Oribitrap high-resolution mass spectrometer (Thermo Fisher Scientific, Bremen, Germany) equipped with a heated electrosprayer for ionization (HESI) was connected in-line with the UPLC system. The mass spectrometer was operated in both negative and positive modes. The ESI parameters were set as follows: spray voltage: +3.5 KV/–2.8 KV; atomization temperature: 350 °C; sheath gas pressure: 50 arb; aux gas pressure: 12.5 arb; capillary temperature: 350 °C; S-lens RF level: 50 V; resolution: MS full scan 70,000 full widths at half maximum (FWHM), MS/MS 17,500 FWHM; scan range: *m*/*z* 100–1500; scanning mode, full scan to data-dependent MS/MS (intensity threshold 800,000). An external calibration for mass accuracy was performed before the analysis according to the manufacturer’s guidelines. All data collected in profile mode were acquired and processed using Thermo Xcalibur 3.0 software. A blank sample barely containing the solvent was added after every 12 samples to attempt to elute residuals or other impurities, which were retained by the chromatography column in the previous spectrum records.

### 4.6. Analysis of Gut Microbiota Composition after Fermentation with TFs

The gut microbiota in the fermentation solution was collected by centrifugation at 10,000× *g* for 5 min, and the genomic DNA was extracted from each sample using the QiAamp Fast D.N.A. Stool Mini Kit (Qiagen, Hilden, Germany), according to the manufacturer’s instructions. The microbial community composition was analyzed by full-length 16S rRNA gene amplicon sequencing carried out by Novogene, Beijing, China. The full V1–V9 region of the bacterial 16S rRNA gene was amplified using the universal primer set 27 F (5′-AGAGTTTGATCCTGGCTCAG-3′) and 1492R (5′-GNTACCTTGTTACGACTT-3′). The PCR products were purified in identical quantities and sequenced on the PacBio platform. Lima was used to distinguish the data of each sample according to the barcode sequence. SSR filtering was carried out and Software Cutadapt (4.1) was applied to remove primers. After being quality filtered, the raw reads were clustered into operational taxonomic units (OTUs) with 97% sequence similarity by UPARSE. The representative sequence for each species OTU was then annotated against the Silva S.S.U. rRNA database with Mothur. The relative abundance of each OTU across all samples was calculated and used for further data mining.

### 4.7. Metabolic Profiling Analysis Based on UPLC-Orbitrap MS/MS 

Based on previous research on the cleavage patterns of flavone and a literature review [33,34,35,36], we studied the fermented metabolites of TFs in vitro by human fecal microbiota, utilizing UPLC-Q-Orbitrap MS/MS coupled with multiple post-acquisition data-mining methods. The identification of the metabolites was performed in light of the following five points: (1) Online data were acquired by full-scan/dd MS2 mode, and the matching fragment information of simultaneous primary and secondary mass spectra and all the possible metabolites of TFs were monitored by metabolite templates that combined a mass defect filter with a background subtraction model in Compound Discovery (CD) 3.0 software. (2) Various data-mining tools containing high-resolution extracted ion chromatograms (HREICs), diagnostic product ions (DPIs) and neutral loss fragments (NLFs), were proposed according to the mass fragmentation behaviors of reference standards, and were applied to seek possible metabolites of TFs. (3) The structures of metabolites were clarified based on accurate mass datasets, parent drug cleavage patterns of TFs, and related drug biotransformation information. (4) As an essential parameter, Clog *p* values predicted by ChemDraw 14.0 were used to discriminate between metabolite isomers, as metabolites with larger Clog *p* values are usually eluted more slowly in reverse-phase chromatographic systems. (5) Finally, the inferred metabolites were verified by comparing their retention times and mass data with those of the standards and the reported literature.

### 4.8. Evaluation of Antioxidant Activity 

DPPH radical scavenging activity was assessed as described previously with some modifications [37]. Briefly, a 50 μL diluted sample solution was mixed with 150 μL of a DPPH working solution (0.20 mmol/L) in a 96-well microplate. Then, after the solution was incubated for 30 min in the dark at room temperature, the absorbance was measured at 517 nm, the 70% (*v*/*v*) CAN solution was used instead of the sample as a negative control (Ac), and DPPH% was calculated following the manufacturer’s instructions. The ABTS radical scavenging activity was measured using a previously described method with some modifications [37]. A total of 20 μL of the sample with various concentrations was mixed with 180 μL of an ABTS^+^ working solution in a 96-well plate, and then the absorbance was measured at 734 nm after the solution was incubated for 6 min in the dark at 37 °C. ABTS% was calculated using the equation provided by the manufacturer.

### 4.9. Data Analysis

Raw LC-MS/MS data were acquired using Thermo Xcalibur software (Version 3.0) and then processed using Thermo CD software (Version 3.0). Data were expressed as mean ± standard deviation (SD) and evaluated by Student’s *t*-test, and a value of *p* < 0.05 was considered statistically significant. Quantitative results were illustrated with GraphPad Prism software (Version 7.00).

## 5. Conclusions

In summary, this study investigated the human-fecal-microbiota-mediated metabolites of TFs and their effect on TFs’ antioxidant capacity. By utilizing UPLC-Orbitrap-MS/MS for metabolite identification, the study found that the human fecal microbiota metabolized TFs into various metabolites, including eight TF derivatives and nine phenolic catabolites. Further, the study also confirmed that TF1 can be oxidized in vitro to form a quinone structure on the benzotropolone moiety, which may further generate oxidized theanaphthoquinone (TNQ) metabolites. However, the specific metabolic pathways involved in this process require further study. In addition, the TFs were found to undergo a series of microbiota-mediated biotransformations, potentially linking the fermentation process with the accumulation of bacteria such as *Parabacteroides_distasonis*, *Flavonifractor_plautii*, *Bacteroides_uniformis*, *Eubacterium_ramulus*,and *Bacteroides_thetaiotaomicron*, which may correspond to metabolic pathways. Additionally, the antioxidant capacity of the TFs was found to be enhanced after their fermentation by the gut microbiota, which may have been related to the metabolites generated in the process. The present findings offer new insights into our understanding of the microbiota-mediated biotransformations and antioxidant capacity of TFs in the human gut.

## Figures and Tables

**Table 1:** UPLC-Q-Orbitrap-MS/MS-based identification of the microbial metabolites of TFs by human fecal fermentation in vitro.

No.	RT.	Tentative Identification	Formula	Theoretical Mass *m*/*z*	Experimental Mass *m*/*z*	Error (ppm)	MS/MS Fragments
M0	11.78	Theaflavine-3,3′-digallate (TF3) ^a^	C_43_H_32_O_20_	868.14869	868.14860	2.18	125.02446, 169.01471, 241.05067, 137.02467, 401.06854
M1	11.5	Theaflavin-3-gallate (TF2A) ^a^	C_36_H_28_O_16_	716.13828	716.13813	−0.21	125.02444, 169.01422, 241.05037, 137.02438, 269.04541
M2	11.71	Theaflavin-3′-gallate (TF2B) ^a^	C_36_H_28_O_16_	716.13828	716.13837	0.13	125.02448, 169.01419, 241.05066, 137.02434, 269.04565
M3	11.28	Theaflavin (TF1) ^a^	C_29_H_24_O_12_	564.12732	564.12598	−2.38	241.05074, 125.02456, 137.02448, 169.01471, 201.05573, 269.04507
M4	11.24	Theaflavin quinone (TQ)	C_29_H_22_O_12_	562.11057	562.11048	−0.16	125.02420, 137.02396, 257.04355, 229.04915
M5	11.05	Theanaphthoquinone (TNQ)	C_28_H_22_O_11_	534.11675	534.11658	−0.32	137.02464, 125.02448, 349.07263, 165.01912, 241.04950
M6	9.1	Dihydro-theaflavin (DH-TF1)	C_29_H_26_O_12_	566.14297	566.14234	−1.11	92.59178, 125.02444, 137.02446, 229.05040, 201.05640, 241.05029
M7	9.16	Dihydro-theaflavin (DH-TF1)	C_29_H_26_O_12_	566.14297	566.14320	0.41	92.59232, 125.02444, 137.02441 165.01921, 201.05542, 229.05046
M8	8.71	Tetrahydro-theaflavin (TH-TF1)	C_29_H_28_O_12_	568.15862	568.15693	−2.97	nd
M9	3.48	Gallic acid ^a^	C_7_H_6_O_5_	170.02207	170.02209	0.53	169.01416, 125.02441, 97.02950
M10	3.60	Pyrogallol ^a^	C_6_H_6_O_3_	126.03224	126.03237	1.03	125.02447, 97.02958, 81.03468
M11	8.69	5-(3′,5′-Dihydroxyphenyl)-γ-valerolactone	C_11_H_12_O_4_	208.0741	208.07407	−0.14	207.06631, 163.07660, 123.04533, 122.03704, 81.0346
M12	9.34	5-(3′,4′-Dihydroxyphenyl)-γ-valerolactone	C_11_H_12_O_4_	208.0741	208.07398	−0.57	163.07663, 207.06635, 122.03751, 81.0347
M13	9.81	5-(Dihydroxyphenyl)-γ-valeric acid (DiHPVA)	C_11_H_14_O_4_	210.08975	210.08910	−3.09	123.08167, 165.09203, 81.03470, 147.08159, 191.07146
M14	8.81	3-(3′,4′-Dihydroxyphenyl) propanoic acid ^a^	C_9_H_10_O_4_	182.05845	182.05843	−0.11	112.98575, 92.99384, 136.98364, 181.09854
M15	11.10	Phenylacetic acid ^a^	C_8_H_8_O_2_	136.05297	136.05312	1.11	91.05544
M16	9.40	3-Hydroxyphenylacetic acid ^a^	C_8_H_8_O_3_	152.04789	152.04777	−0.73	107.05035, 151.04008
M17	7.84	4-Hydroxybenzoic acid ^a^	C_7_H_6_O_3_	138.03224	138.03210	−1.01	93.03462, 95.05029, 137.06078

^a^: Confirmation in comparison with authentic standards; RT: retention time; nd: not detected.

## Data Availability

Not applicable.

## Supplementary Materials

The following supporting information can be downloaded at https://www.mdpi.com/article/10.3390/molecules28155871/s1: Figure S1: Total ion chromatograms (TIC) of control 1 using human fecal suspension (HFS) and medium (A), control 2 using TFs incubated for 0 h by HFS(B), and TF samples mixed at different time points after fermentation (2, 4, 8, 12, 24, and 48 h) by HFS(C); Figure S2: The MS2 spectrum and cleavage pathways of M5; Figure S3: The MS/MS spectrum of all identified metabolites; Table S1. The peak areas of metabolites detected fermentation broth at different fermentation times at 0, 2, 4, 8, 12, 24, and 48 h, respectively.

## Sample Availability

The samples of some compounds in this study are available from the corresponding author upon reasonable request.
