# Supplementary material for: Microbial-Transferred Metabolites of Black Tea Theaflavins by Human Gut Microbiota and Their Impact on Antioxidant Capacity
Source: Molecules. 2023 Aug 4;28(15):5871. doi: 10.3390/molecules28155871 (PMC10420933; doi:10.3390/molecules28155871)
Supplement: Supplementary file 1 [file molecules-28-05871-s001.zip › molecules-2488492-supplementary.pdf]

molecules

MDPI

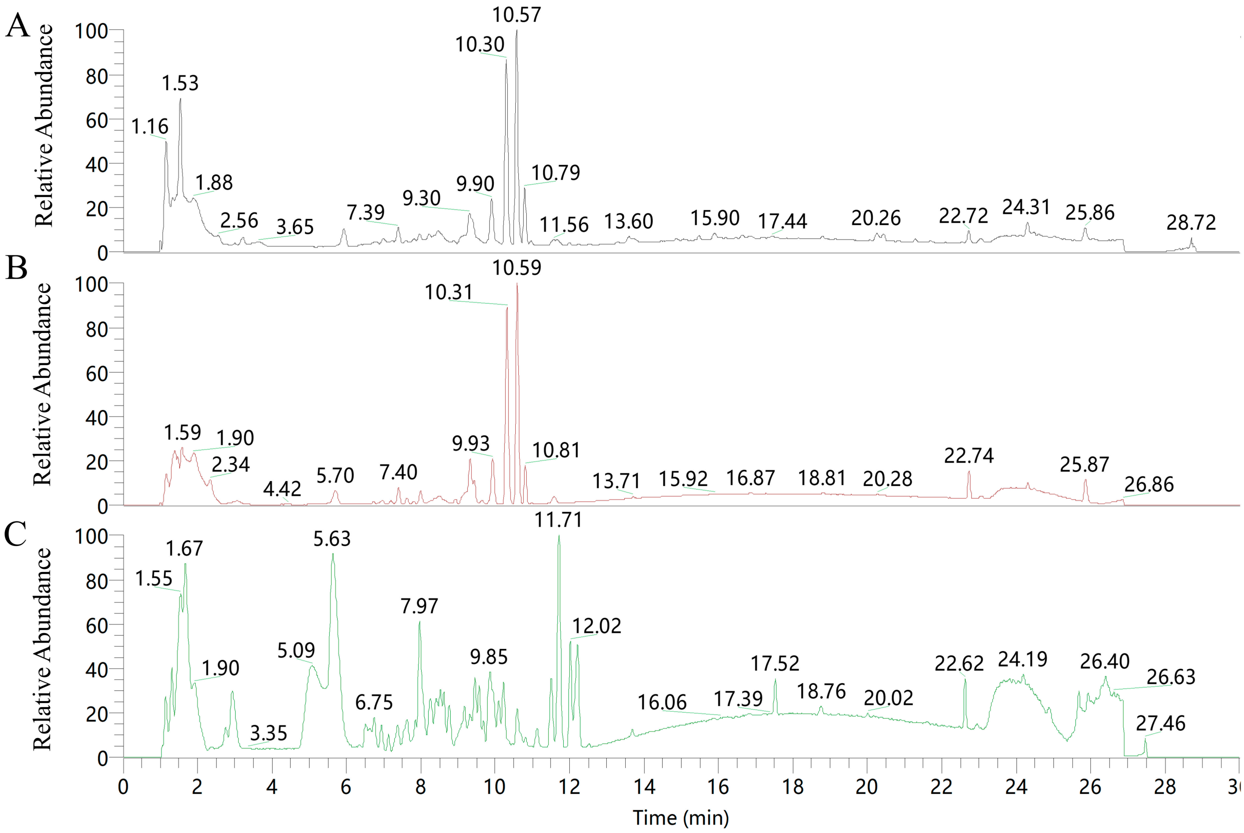

Figure S1. Total ion chromatograms (TIC) of control 1 using human fecal suspension (HFS) and medium (A), control 2 using TFs incubated for 0h by HFS(B), and TFs samples mixed at different time points after fermentation by HFS(C).

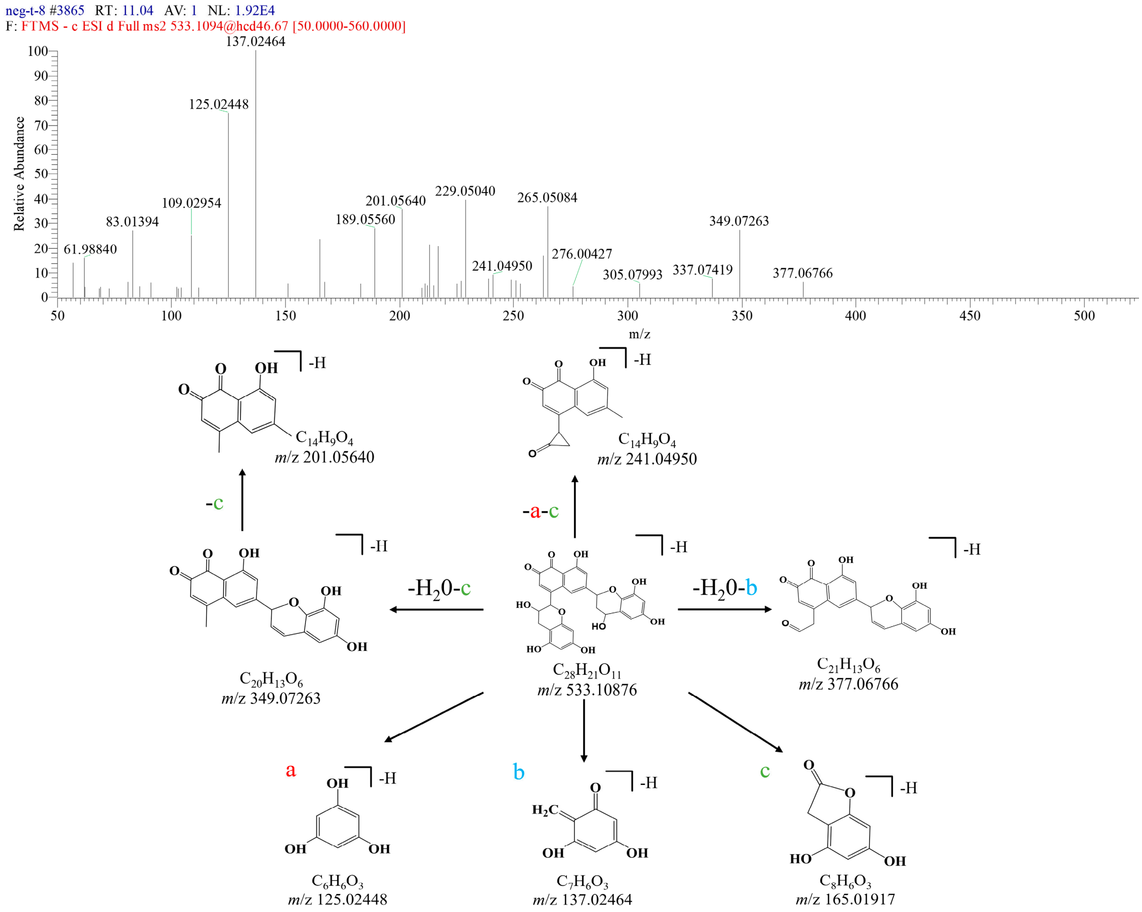

Figure S2. The MS2 spectrum and probable fragmentation pathways of M5.

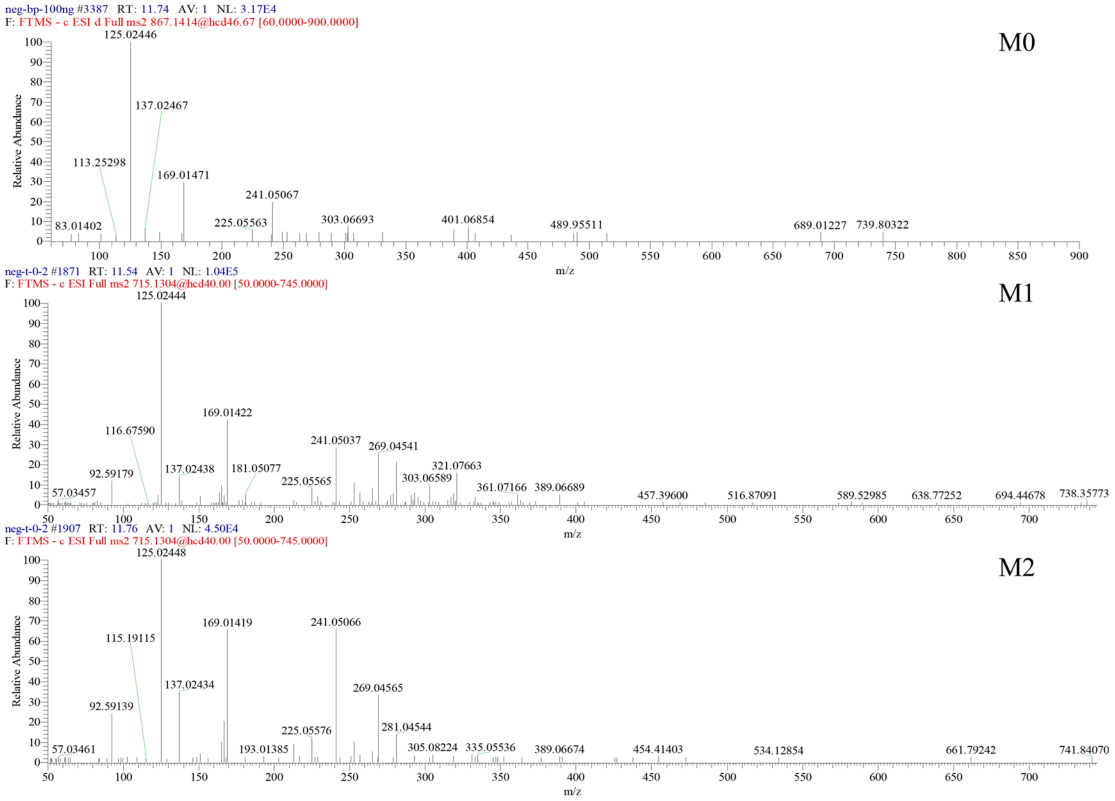

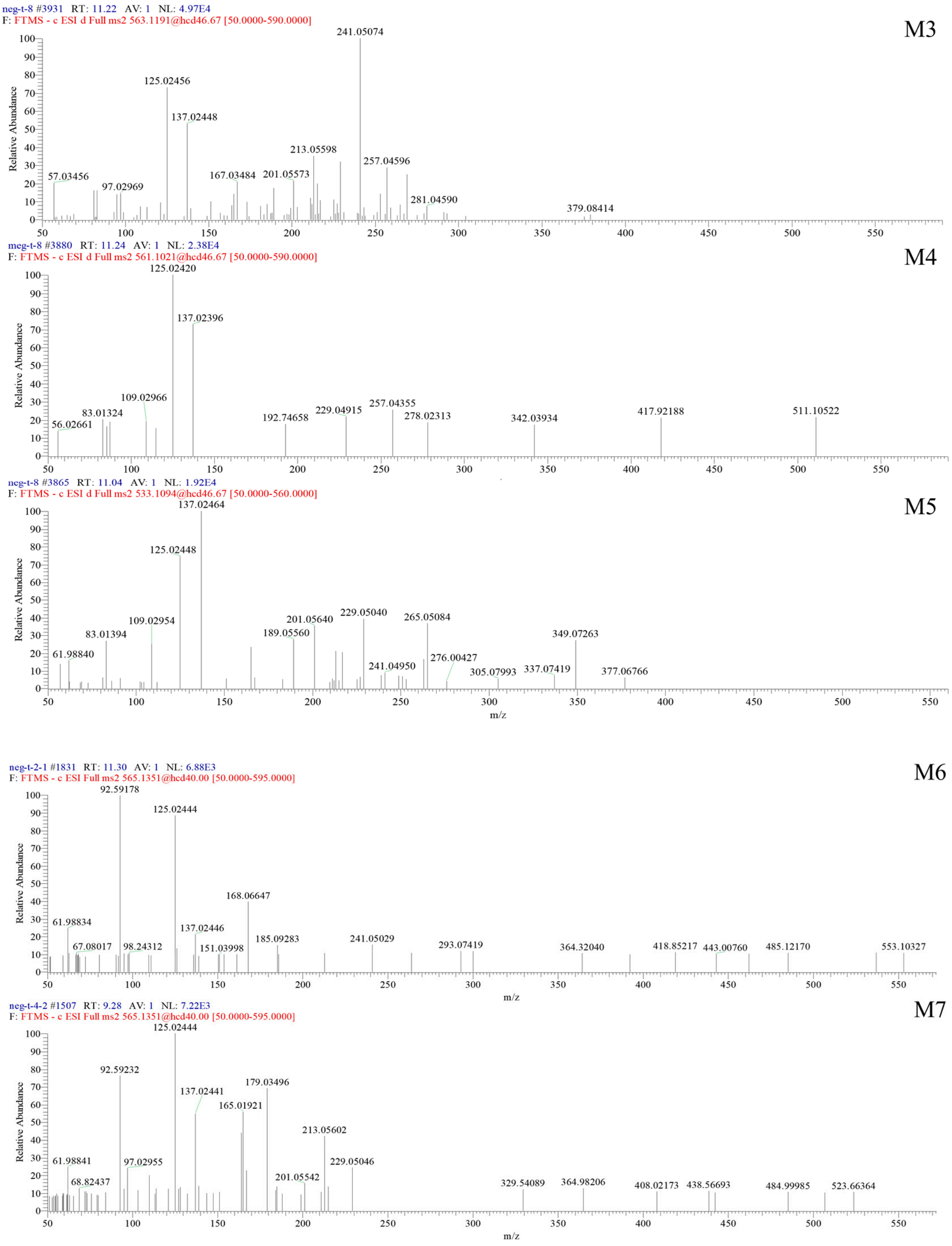

neg-t-8 #3931 RT: 11.22 AV: 1 NL: 4.97E4
F: FTMS - c ESI d Full ms2 563.1191@hcd46.67 [50.0000-590.0000]
M3
241.05074
125.02456
137.02448
213.05598
257.04596
57.03456
97.02969
167.03484
201.05573
281.04590
379.08414
Relative Abundance
meg-t-8 #3880 RT: 11.24 AV: 1 NL: 2.38E4
F: FTMS - c ESI d Full ms2 561.1021@hcd46.67 [50.0000-590.0000]
M4
125.02420
137.02396
109.02966
83.01324
56.02661
192.74658
229.04915
257.04355
278.02313
342.03934
417.92188
511.10522
neg-t-8 #3865 RT: 11.04 AV: 1 NL: 1.92E4
F: FTMS - c ESI d Full ms2 533.1094@hcd46.67 [50.0000-560.0000]
M5
137.02464
125.02448
109.02954
83.01394
61.98840
201.05640
229.05040
265.05084
189.05560
349.07263
241.04950
276.00427
305.07993
337.07419
377.06766
m/z
neg-t-2-1 #1831 RT: 11.30 AV: 1 NL: 6.88E3
F: FTMS - c ESI Full ms2 565.1351@hcd40.00 [50.0000-595.0000]
M6
92.59178
125.02444
168.06647
61.98834
137.02446
67.08017
98.24312
151.03998
185.09283
241.05029
293.07419
364.32040
418.85217
443.00760
485.12170
553.10327
neg-t-4-2 #1507 RT: 9.28 AV: 1 NL: 7.22E3
F: FTMS - c ESI Full ms2 565.1351@hcd40.00 [50.0000-595.0000]
M7
125.02444
92.59232
179.03496
137.02441
165.01921
213.05602
61.98841
97.02955
229.05046
68.82437
201.05542
329.54089
364.98206
408.02173
438.56693
484.99985
523.66364

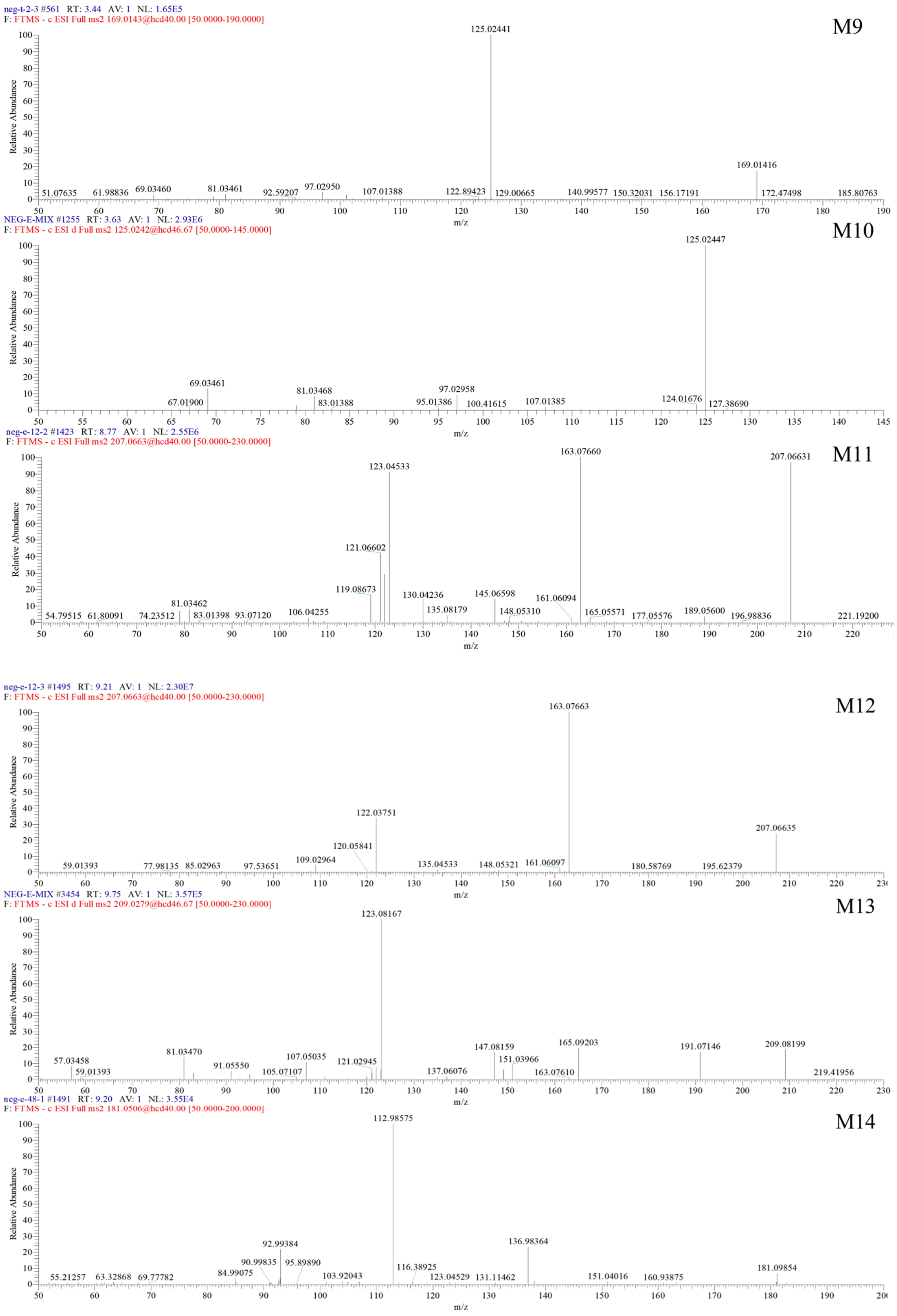
neg-t-2-3 #561 RT: 3.44 AV: 1 NL: 1.65E5
F: FTMS - c ESI Full ms2 169.0143@hcd40.00 [50.0000-190.0000]
M9
125.02441
169.01416
51.07635 61.98836 69.03460 81.03461 92.59207 97.02950 107.01388 122.89423 129.00665 140.99577 150.32031 156.17191 172.47498 185.80763
Relative Abundance
m/z
NEG-E-MIX #1255 RT: 3.63 AV: 1 NL: 2.93E6
F: FTMS - c ESI d Full ms2 125.0242@hcd46.67 [50.0000-145.0000]
M10
125.02447
69.03461 67.01900 81.03468 83.01388 97.02958 95.01386 100.41615 107.01385 124.01676 127.38690
Relative Abundance
m/z
neg-e-12-2 #1423 RT: 8.77 AV: 1 NL: 2.55E6
F: FTMS - c ESI Full ms2 207.0663@hcd40.00 [50.0000-230.0000]
M11
163.07660
207.06631
123.04533
121.06602
119.08673
130.04236 135.08179 145.06598 148.05310 161.06094
54.79515 61.80091 74.23512 81.03462 83.01398 93.07120 106.04255 165.05571 177.05576 189.05600 196.98836 221.19200
Relative Abundance
m/z
neg-e-12-3 #1495 RT: 9.21 AV: 1 NL: 2.30E7
F: FTMS - c ESI Full ms2 207.0663@hcd40.00 [50.0000-230.0000]
M12
163.07663
122.03751
207.06635
120.05841
59.01393 77.98135 85.02963 97.53651 109.02964 135.04533 148.05321 161.06097 180.58769 195.62379
Relative Abundance
m/z
NEG-E-MIX #3454 RT: 9.75 AV: 1 NL: 3.57E5
F: FTMS - c ESI d Full ms2 209.0279@hcd46.67 [50.0000-230.0000]
M13
123.08167
147.08159 165.09203 191.07146 209.08199
57.03458 59.01393 81.03470 91.05550 105.07107 107.05035 121.02945 137.06076 151.03966 163.07610 219.41956
Relative Abundance
m/z
neg-e-48-1 #1491 RT: 9.20 AV: 1 NL: 3.55E4
F: FTMS - c ESI Full ms2 181.0506@hcd40.00 [50.0000-200.0000]
M14
112.98575
92.99384
136.98364
90.99835 95.89890 84.99075
55.21257 63.32868 69.77782 103.92043 116.38925 123.04529 131.11462 151.04016 160.93875 181.09854
Relative Abundance
m/z

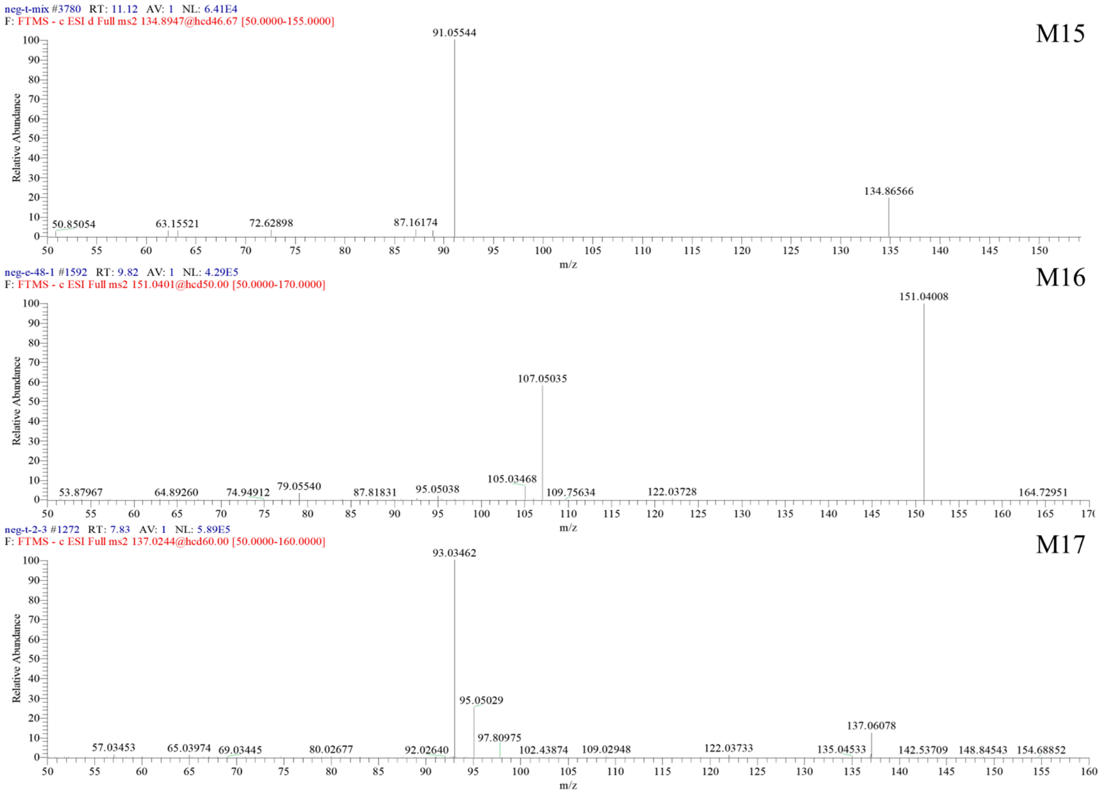

Figure S3. The MS/MS spectrum of all metabolites.

Table S1. The peak areas of metabolites detected fermentation broth at different fermentation times at 0, 2, 4, 8, 12, 24, 48 h, respectively.

	**0 h**	**2h**	**4 h**	**8 h**	**12 h**	**24 h**	**48 h**
	Peak area	Peak area	Peak area	Peak area	Peak area	Peak area	Peak area
**M0**	8.46E+07	6.61E+07	5.42E+07	2.63E+06	4.80E+04	1.48E+04	1.17E+04
**M1**	4.42E+06	6.41E+07	9.63E+07	161456-	4.53E+05	9.38E+04	3.24E+06
**M2**	1.89E+06	2.13E+07	2.00E+07	1.64E+06	3.23E+05	2.18E+05	1.88E+04
**M3**	5.08E+06	4.38E+07	3.50E+07	4.05E+06	7.35E+05	3.63E+05	2.46E+05
**M4**	-	5.82E+05	8.45E+05	1.43E+06	1.41E+03	-	-
**M5**	-	9.24E+05	1.58E+06	4.29E+06	-	-	-
**M6**	-	3.83E+04	3.07E+05	1.82E+06	-	-	-
**M7**	-	6.08E+04	1.04E+05	6.29E+05	-	-	-
**M8**	-	-	-	2.67E+04	-	-	-
**M9**	-	1.48E+06	1.56E+06	2.55E+04	-	-	-
**M10**	-	1.87E+06	5.35E+06	9.50E+03	-	-	-
**M11**	-	4.67E+04	5.74E+04	8.96E+04	6.57E+04	2.60E+05	2.88E+05
**M12**	-	5.87E+05	7.38E+05	6.61E+05	4.20E+03	5.61E+03	4.16E+03
**M13**	-	-	-	-	2.32E+04	9.32E+04	2.74E+05
**M14**	-	1.43E+05	2.10E+05	1.45E+05	4.10E+04	3.36E+04	1.07E+05
**M15**	-	-	-	1.87E+05	3.08E+05	6.81E+05	1.23E+06
**M16**	-	-	-	5.60E+04	6.47E+04	2.10E+05	5.22E+05
**M17**	3.73E+05	3.02E+05	5.91E+05	7.77E+05	9.67E+05	1.10E+06	1.19E+06
